# Ethanol Metabolism and Melanoma

**DOI:** 10.3390/cancers15041258

**Published:** 2023-02-16

**Authors:** Zili Zhai, Takeshi Yamauchi, Sarah Shangraw, Vincent Hou, Akiko Matsumoto, Mayumi Fujita

**Affiliations:** 1 Department of Dermatology, University of Colorado Anschutz Medical Campus, Aurora, CO 80045, USA; 2 Department of Social Medicine, School of Medicine, Saga University, Saga 849-8501, Japan; 3 Department of Immunology and Microbiology, University of Colorado Anschutz Medical Campus, Aurora, CO 80045, USA; 4 Department of Veterans Affairs Medical Center, VA Eastern Colorado Health Care System, Aurora, CO 80045, USA

**Keywords:** melanoma, ethanol, acetaldehyde, ethanol metabolism, alcohol dehydrogenase, aldehyde dehydrogenase

## Abstract

**Simple Summary:**

Acetaldehyde (AcAH) is a carcinogenic byproduct of ethanol metabolism. Ethanol-associated malignancies commonly occur in the upper gastrointestinal tract exposed to AcAH after ethanol ingestion. Unexpectedly but true, emerging epidemiological evidence supports a link between alcohol consumption and cutaneous melanoma, suggesting skin exposure to ethanol and AcAH as potential causes of skin cancer. Humans are unavoidably exposed to ethanol and AcAH daily in multiple ways, such as through alcohol consumption, food ingestion, inhalation, skin contact, and bodily microbiota. This review examines the sources of ethanol and AcAH in the skin, their metabolic pathways, and the consequences of dysfunctional ethanol and AcAH metabolizing enzymes, focusing on the role of these factors in melanoma development and progression.

**Abstract:**

Malignant melanoma is the deadliest form of skin cancer. Despite significant efforts in sun protection education, melanoma incidence is still rising globally, drawing attention to other socioenvironmental risk factors for melanoma. Ethanol and acetaldehyde (AcAH) are ubiquitous in our diets, medicines, alcoholic beverages, and the environment. In the liver, ethanol is primarily oxidized to AcAH, a toxic intermediate capable of inducing tumors by forming adducts with proteins and DNA. Once in the blood, ethanol and AcAH can reach the skin. Although, like the liver, the skin has metabolic mechanisms to detoxify ethanol and AcAH, the risk of ethanol/AcAH-associated skin diseases increases when the metabolic enzymes become dysfunctional in the skin. This review highlights the evidence linking cutaneous ethanol metabolism and melanoma. We summarize various sources of skin ethanol and AcAH and describe how the reduced activity of each alcohol metabolizing enzyme affects the sensitivity threshold to ethanol/AcAH toxicity. Data from the Gene Expression Omnibus database also show that three ethanol metabolizing enzymes (alcohol dehydrogenase 1B, P450 2E1, and catalase) and an AcAH metabolizing enzyme (aldehyde dehydrogenase 2) are significantly reduced in melanoma tissues.

## 1. Introduction

Melanoma is the most serious type of skin cancer, with an increasing incidence worldwide [1,2]. Cumulative evidence indicates that the risk of melanoma correlates with genetic factors [3,4], personal lifestyles [5,6], and phenotypic risk factors that reflect gene/personal lifestyle interactions [7,8]. Individual lifestyle factors associated with melanoma risk include UV exposure, cigarette smoking, alcohol use, overweight and obesity, poor diet, environmental pollution, and stress [5,6]. Recently, we summarized epidemiological data on the association between alcohol consumption and cutaneous melanoma [9]. Nearly half (14 out of 29) of the studies on the relationship between alcohol consumption and melanoma, including 10 cohort and 19 case-control studies, have shown a positive correlation, while only 2 showed a negative correlation. Further, of 20 studies assessing alcohol dose effects, 13 (65%) demonstrated an association between alcohol dose and melanoma risk [9]. These associations became stronger in multiple meta-analyses with larger sample sizes [10,11,12,13]. A systematic meta-analysis by Gandini et al. found that individuals in the highest category of recent alcohol intake had a 30% increased risk of melanoma compared to those in the lowest category, and a nearly two-fold increased risk of melanoma was found with cumulative alcohol consumption [14].

Approximately 4% of cancer cases worldwide are caused by alcohol consumption [15]. The main culprit for this is acetaldehyde (AcAH), an immediate metabolite of ethanol. AcAH is a mutagen and carcinogen implicated in a wide range of cancers by forming adducts with proteins and DNA and disrupting cellular functions [16,17]. However, ethanol and AcAh are derived not just from alcohol drinking but from various sources, some of which exist naturally [18,19]. In fact, our skin is exposed to ethanol and AcAH every day, regularly at extremely low and safe levels [18,19,20]. While our skin, like the liver, is equipped with ethanol metabolism mechanisms to reduce the concentration of ethanol and AcAH [18,20], dysfunction of these ethanol and AcAH metabolizing enzymes in the skin may greatly influence the skin biology and increase the risk of ethanol/AcAH-associated skin diseases. 

This review summarizes various sources of ethanol and AcAH in the skin and explains how ethanol metabolism can affect an individual’s sensitivity threshold to AcAH carcinogenesis. 

## 2. Exposure to Ethanol and AcAH

Humans are in a chemical and toxicological environment [21] and are exposed to ethanol and AcAH in many ways. Once in the bloodstream, ethanol and AcAH can reach many organs and tissues, including the skin [22]. The skin is also directly exposed to alcohols and aldehydes from natural chemicals or industrialized products [19,20]. 

### 2.1. Sources of Ethanol

**Figure 1:**
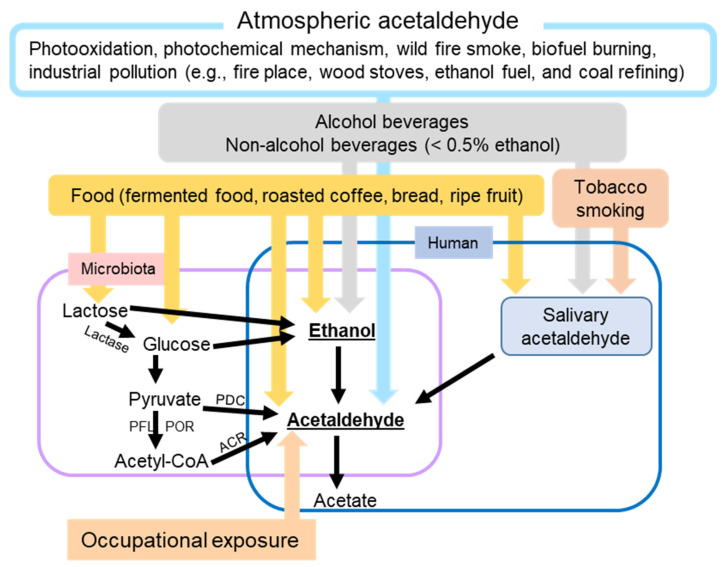
Schematic overview of the major factors leading to human skin exposure to ethanol and/or acetaldehyde. ACR, acetyl-CoA reductase; PDC, pyruvate decarboxylase; PFL, pyruvate formate-lyase; POR, pyruvate ferredoxin oxidoreductase.

Ethanol is not only the active ingredient of alcoholic beverages (beers, wines, and spirits) but also is a ubiquitous substance from various sources (Figure 1). It is one of the main indoor and outdoor pollutants [19]. In addition to alcoholic beverages and air pollutants, non-alcoholic beverages on the market can contain as much as 0.5% ethanol [23]. Certain herbal medicines, including those used to treat coughs, colds, and gastrointestinal (GI) diseases, are also sources of ethanol [24]. Furthermore, many foods contain ethanol, which is produced from sugar through fermentation. Examples include fermented foods (i.e., bread, yogurt, vinegar, and kimchi), preservatives, bakery products, fruit, and fruit juices [25]. Brewers and bakers use yeast to make a variety of alcoholic beverages and expand the dough. 

Even without the exogenous ethanol intake mentioned above, our bodies contain low levels of ethanol. Baseline ethanol levels in the blood and breath can reach 0.02–0.15 mg/dL and 0.07–0.39 mg/L, respectively, without consuming alcohol [26]. These low levels of ethanol are generated by microbial fermentation. Fermenting yeasts such as *Saccharomyces* and *Candida* (*C.*) and fermenting bacteria such as *Zymomonas mobilis* and *Sarcina ventriculi* are present in our oral cavity and digestive tract. These microbiotas use anaerobic respiration to convert non-ethanol, carbohydrate-rich foods such as glucose and lactose to ethanol by fermentation in the oral cavity, GI system, or urinary system [27]. This microbial ethanol production is particularly interesting in certain medical conditions.

In auto-brewery syndrome (ABS), also known as gut fermentation syndrome, microbiota-derived ethanol concentrations in the body are comparable to those produced by directly consuming alcoholic beverages [28,29]. In a study conducted by Malik et al., the blood alcohol concentration (BAC) in an ABS patient reached 400 mg/dL [30], which is comparable to a BAC that can cause respiratory depression, coma, and death [31]. The species causing the ABS include *Klebsiella pneumoniae*, *C. albicans*, *C. glabrata*, *Saccharomyces cerevisiae*, *C. intermedia*, *C. parapsilosis*, and *C. kefyr* [32]. ABS is a rare condition. This syndrome could be underdiagnosed, as the symptoms may be mood changes, delirium, and brain fog or mimic a food allergy [30,32,33]. Triggers of this ABS include meals high in carbohydrates, psychological stress, and reduced dietary intake. ABS may also be related to pre-existing conditions, including a history of antibiotic use and comorbidities, such as type 2 diabetes, obesity, liver cirrhosis, and Crohn’s disease [27].

Ethanol can also be produced from AcAH by microbial reduction [34]. 

### 2.2. Sources of AcAH

Similar to ethanol exposure, human exposure to AcAH is not only from alcohol consumption but also from other diverse sources (Figure 1). AcAH can be produced endogenously in any tissue with high ethanol metabolizing enzyme activity [35]. Bodily AcAH may come from oral and gut microbes that metabolize ethanol to AcAH. Salivary AcAH levels reached up to 140 µM after ingesting a moderate amount of ethanol (0.5 g/kg body weight) [35]. Long-term exposure to locally produced AcAH in saliva may explain the higher risk of upper GI cancers in heavy drinkers [35]. 

The atmospheric AcAH are from photochemical production, net ocean emissions, biogenic emissions, biomass burning emissions, and anthropogenic emissions, accounting for 60%, 27%, 11%, 1.6%, and < 1% of global AcAH production, respectively [36]. The largest source is the photo-oxidation of volatile organic compounds such as alkanes and alkenes [36]. The second-largest source is water bodies that degrade dissolved organic compounds through the photochemical mechanism and emit AcAH into the atmosphere [37]. Most plant cells and some microorganisms use anaerobic respiration to break down glucose to AcAH and release carbon dioxide (biogenic emissions). Biomass burning emissions are from wildfire smoke and biofuel burning. Another source of AcAH includes urban and industrial pollution, such as residential fireplaces, woodstoves, ethanol fuel, vehicle exhaust fumes, coal refining, and waste processing. Therefore, like ethanol, AcAH is a common noxious environmental pollutant [19]. Nevertheless, sinks of atmospheric AcAH include reaction with hydroxyl radical, photolysis, and wet and dry deposition, leading to an overall atmospheric lifetime for AcAH of approximately 20 h [36,38]. 

AcAH sources also include occupational exposure. Individuals may be at risk of higher AcAH exposure when working in gas stations [39], transportation vehicles [40], waterpipe café [41], bakeries [42], and beauty salons [43], as well as in industries using AcAH as a solvent for perfumes, polyester resins, acetic acid, mirror silvering, tanning leather, denaturing alcohol, fuel compositions, gelatin fiber hardeners, glue and casein products, paper, cosmetics, aniline dyes, plastics, and synthetic rubber [44]. 

AcAH is also contained in various foods (e.g., fermented food, roasted coffee, bread, and ripe fruit) and beverages and is used as a flavoring agent and a preservative for fruits and fish [45,46]. 

Furthermore, AcAH is a byproduct of tobacco smoking [47]. When coupled with nicotine, AcAH has been shown to increase the addictive potential of smoking [48,49]. Nieminen et al. reported that the concentration of AcAH in saliva remains as high as 261 μM with one cigarette, which is higher than the AcAH concentration after drinking high-concentration (40%) ethanol for a short period [47]. Smoking can increase AcAH production from ethanol in saliva by 60–75%; for heavy drinking, the increase in AcAH is up to 100% [47]. 

Another source of AcAH is pyruvate, an important energy source produced during glycolysis. In anaerobic conditions, yeast use pyruvate decarboxylase (PDC) to convert pyruvate to AcAH, and *C. albicans*, an opportunistic pathogenic yeast, has been shown to contribute to oral squamous cell carcinoma progression by producing high levels of AcAH from glucose under low oxygen conditions [50,51]. Some bacteria also use PDC to convert pyruvate to AcAH. However, as a common pathway in bacteria, pyruvate is first decarboxylated to acetyl-CoA by pyruvate ferredoxin oxidoreductase and/or pyruvate formate-lyase. Acetyl-CoA is then converted into AcAH by acetylating acetyl-CoA reductase in bacteria [52]. Since the enzymes to produce AcAH from pyruvate have not been reported in humans, pyruvate-derived AcAH is likely produced from bodily microbiota rather than human cells.

Collectively, ethanol and AcAH are part of our life. The skin is exposed to these toxic metabolites through the air, water, land, smoke, food, and bodily microbiota, among other means. Since ethanol and AcAH are readily degraded in the environment or metabolized in the body, the anticipated skin exposure levels are very low or safe. However, when the body’s metabolic process becomes dysfunctional, high levels of ethanol and AcAH can cause health consequences, including skin diseases. 

## 3. Ethanol Metabolism and Its Contribution to Human Diseases

Due to their ubiquitous nature, ethanol and AcAH can affect our health daily, particularly in industrialized countries [19]. In this context, ethanol metabolism is important since it directly determines the fate of ethanol and AcAH in the body, with varying degrees of biological consequences. 

The first-pass metabolism of ethanol after its ingestion occurs in the GI mucosa and flora before it reaches the bloodstream and then in the liver, where most ethanol metabolizing enzymes are present [53,54]. Overall, over 90% of ingested ethanol is metabolized, and the remainder is excreted through breath (1–3%), urine (1–3%), and sweat (traces) without modification [55,56]. As our bodies, including the skin, are exposed to ethanol [18,20], humans have evolved to harbor a set of metabolic detoxifying enzymes to reduce the toxic effects of alcohols, aldehydes, and other xenobiotics from various sources. This chapter will explain the roles of each enzyme, its expression, and the potential consequences when the enzyme becomes dysfunctional, particularly its carcinogenic effects.

### 3.1. Ethanol Metabolizing Enzymes and Their Impacts on Humans

**Figure 2:**
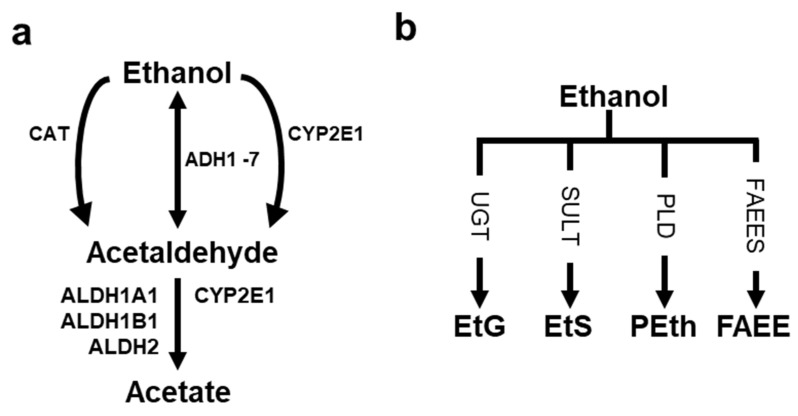
Schematic overview of ethanol metabolism. Ethanol can be metabolized by oxidative (**a**) or non-oxidative (**b**) pathways. ADH, alcohol dehydrogenase; ALDH, aldehyde dehydrogenase; CAT, catalase; CYP2E1, cytochrome P450 2E1; EtG, ethyl glucuronide; EtS, ethyl sulfate; FAEE, fatty acid ethyl ester; FAEES, fatty acid ethyl ester synthase; PEth, phosphatidyl ethanol; PLD, phospholipase D; SULT, sulfotransferase; UGT, UDP-glucuronosyltransferase. Modified from [9].

The main pathway of ethanol metabolism is oxidation into the highly reactive AcAH [57] (Figure 2a). The predominant enzyme that oxidizes ethanol to AcAH is cytosolic alcohol dehydrogenase (ADH) [58]. Other enzymes that convert ethanol to AcAH are microsomal cytochrome P450 2E1 (CYP2E1) and peroxisomal catalase (CAT) [54,59]. 

There are seven isoforms of ADH [60]. So far, all of them are known to participate in ethanol oxidation except ADH6 [61]. Although ADH families have a limited distribution in human tissues, primarily in hepatocytes, they are also found in the GI tract and certain tissues, including the skin epidermis and dermis [18,58]. 

ADH1A, ADH1B, and ADH1C (referred to as ADH1-3) play a dominant role in the oxidative metabolism of ethanol in the liver after low to moderate alcohol consumption [60,61]. Genetic polymorphisms with physiological significance occur in *ADH1B* and *ADH1C* [61]. The common ADH1B*2 allele in East Asia (75%) and the relatively common ADH1B*3 allele in Eastern Africa (10-30%) [61] display quick ethanol turnover, leading to rapid accumulation of AcAH following ethanol intake [62]. A meta-analysis of 18 studies demonstrates that *ADH1B* polymorphisms, particularly rs1229984 Arg47His with a faster metabolic character, increase the risk of bladder cancer, colorectal cancer, and upper aerodigestive tract cancers (tumors of the oral cavity, pharynx, larynx, and esophagus) for alcoholics [63]. On the contrary, the ADH1C*2 allele metabolizes ethanol < 2.5 times slower than the ADH1C*1 allele. Therefore, Caucasians with ADH1C*1/2 heterogeneity have an increased risk for alcohol-related esophageal, hepatocellular, and head and neck cancers due to slow ethanol turnover [64]. Interestingly, a paper reports that these enzymes are also expressed in the epidermis of human skin (foreskin, breast, and abdomen) [18]. 

ADH4, a class II ADH, is active only when large quantities of ethanol are consumed and is expressed almost exclusively in the liver [61]. ADH5, a class III ADH, is ubiquitously expressed and implicated in the first-pass metabolism of ethanol [65,66]. ADH5 has two other main functions: it inactivates formaldehyde and nitric oxide by converting them into formic acid and *S*-nitrosoglutathione, respectively, in a glutathione-dependent manner [67,68]. Formaldehyde, an endogenous byproduct of oxidative demethylation reactions, is genotoxic and carcinogenic, as it cross-links proteins and nucleic acids [69]. Therefore, ADH5 dysfunction in scavenging formaldehyde is linked to defective hematopoiesis and increased leukemia [70]. ADH7, a class III ADH, is expressed in the GI tract endothelial cells and implicated in ethanol’s first-pass metabolism [65].

CYP2E1 constitutes the microsomal ethanol oxidizing system that converts ethanol to AcAH, accounting for around 10% of the total ethanol oxidizing capacity of the liver [54]. CYP2E1 is highly expressed in the liver but is also detectable in extrahepatic tissues, including the lung, kidney, skin, brain, placenta, and testis [71,72]. Although CYP2E1 is critical for oxidizing higher levels of ethanol due to its lower affinity for ethanol than most ADH isoforms, the fact is that CYP2E1 is inducible up to 10-fold by ethanol [60,73]. An alcohol-induced increase in the microsomal pathway contributes to liver pathology due to the generation of reactive oxygen species (ROS) during this reaction [54,57,60], leading to the development of hepatocellular carcinoma [74]. Diet, lifestyle, and physiological factors substantially influence CYP2E1 phenotype. 

Hepatic CAT plays a minimal role in ethanol metabolism. However, blood CAT activity significantly correlates with alcohol consumption [75]. Since its oxidation of ethanol is hydrogen peroxide (H_2_O_2_)-dependent, CAT works more efficiently under elevated ROS levels and oxidative stress following heavy drinking [54]. Since CAT-catalyzed ethanol oxidation occurs in the brain, this gene may impact susceptibility to alcohol dependence [76]. While CAT has a limited role in ethanol clearance, its polymorphism rs1001179 (Cys262Thr) has been shown to increase prostate cancer risk because of increased ROS [77,78]. Several reports have demonstrated that CAT is highly expressed in the skin. CAT activity in the epidermis is more than eight times higher than in the underlying dermis [79] and plays a key role in protecting skin against UV radiation and skin aging [80,81]. In epidermal melanocytes, the expression and activity of CAT are directly related to melanin content, acting synergistically to defend against solar UV damage [82]. Consistent with these reports, reduced CAT levels and increased H_2_O_2_ levels in the skin have been related to vitiligo and xeroderma pigmentosum [83,84,85,86]. 

While a majority of ingested ethanol undergoes oxidative metabolism, a small fraction (0.1–0.2%) can undergo non-oxidative metabolism [56] (Figure 2b), resulting in the enzymatic conjugation of ethanol to endogenous metabolites to form ethyl glucuronide (EtG), ethyl sulfate (EtS), phosphatidylethanol (PEth), and fatty acid ethyl ester (FAEE) [87,88]. EtG and EtS can be retained in the blood (1–3 h), urine (2–4 weeks), and keratinized matrices such as hair (months), and these metabolic products are used as metabolic biomarkers of recent alcohol consumption [89,90]. For example, transdermal sensors have been developed to measure skin concentrations of ethanol or its metabolite EtG after alcohol drinking [91]. The metabolites produced by non-oxidative metabolism are not considered non-toxic. EtG and EtS affect toll-like receptor signaling and reduce energy metabolism [92]. Phospholipase D, an enzyme synthesizing PEth, is involved in keratinocyte differentiation [93]. FAEE interferes with cell signaling pathways and disrupts organelle function, contributing to ethanol toxicity in tissues with a limited oxidative capacity [88].

Additionally, ethanol itself affects the human body. Ethanol can enhance the activity of adenylyl cyclase, one of the main targets of ethanol in the cAMP/protein kinase A (PKA) signaling pathway [94,95]. PKA phosphorylates and dephosphorylates many proteins, mediating various cellular processes, including glucose and lipid metabolism, cell growth, differentiation, and death [96]. 

In conclusion, ethanol metabolizing enzymes and pathways are related to the development of cancers. While most exogenous ethanol is metabolized in the GI tract and liver, a fraction goes to the peripheral tissues. Due to the lack of plasma protein binding, ethanol is readily distributed in all body tissues except fat and bone [60]. Therefore, the skin may have evolved to express ethanol metabolizing enzymes. 

### 3.2. AcAH Metabolizing Enzymes and Their Effects on Humans

Since AcAH is incriminated in pathogenesis by its potent and lasting damage to cellular macromolecules and its mutagenic and carcinogenic effects on DNA, removing AcAH from the body is essential [97]. In the second step of oxidative ethanol metabolism, AcAH is further oxidized to acetate by aldehyde dehydrogenase (ALDH), mainly cytosolic ALDH1A1, mitochondrial ALDH1B1, and mitochondrial ALDH2 (Figure 2a). ALDH1A1 has an affinity for AcAH but metabolizes AcAH less efficiently. ALDH1B1 and ALDH2 have a broad tissue distribution and are catalytically active toward a wide range of aldehyde substrates [20,98,99]. ALDH1B1 shares a 75% peptide sequence homology with ALDH2 [100]. 

ALDH1A1 and ALDH1B1 have limited involvement in AcAH metabolism under physiological conditions, as they have a higher Km for AcAH compared to ALDH2 (with Km values of 180, 55, and 0.2 μM, respectively) [100]. Polymorphisms of *ALDH1A1* and *ALDH1B1* have been identified in Europeans and are associated with alcohol-related phenotypes [101,102].

ALDH2 is involved in oxidizing toxic aldehydes of both exogenous and endogenous sources, including those generated from endoplasmic reticulum stress, oxidative stress, and other stress-inducing conditions [99,103]. Thus, ALDH2 has special physiopathological significance, and its downregulation and inactivation have been associated with many health conditions, including cardiovascular diseases, neurodegenerative diseases, alcohol-related liver disease, and cancer [99]. 

ALDH2 downregulation and inactivation are the results of the complex interplay between genetic susceptibility (e.g., allelic variation, polymorphism, and epigenetics) and multiple environmental factors (e.g., alcohol, smoking, drugs, and high-fat diet) [104]. One of the well-studied *ALDH2* polymorphisms is the ALDH2*2 allele (*rs671*), which results from a single point mutation changing the glutamic acid at the 487th position to a lysine (Glu487Lys of mature protein or Glu504Lys of the precursor protein) [61]. Due to the tetrameric structure of ALDH2 and the dominant inactive phenotype of the variant, individuals can be categorized into three *ALDH2* genotypes. The wild-type ALDH2*1/*1 genotype possesses a normal enzymatic activity, while the heterozygous ALDH2*1/*2 genotype retains approximately 15.6% of normal activity, and the homozygous ALDH2*2/*2 genotype loses total enzyme activity [105]. The ALDH2*2 (either ALDH2*1/*2 or ALDH2*2/*2) mutation is one of the most common genotypes carried by more than 8% of the world population and is frequent in East Asians, with a prevalence of 30–50% [106]. When similar amounts of ethanol are consumed, *ALDH2*-deficient individuals have significantly higher AcAH-derived DNA adducts in their blood than individuals with the wild-type genotype [107]. 

The mechanistic basis behind ALDH2 downregulation without *ALDH2* polymorphisms is complex and multi-factorial: (1) *ALDH2* expression can be epigenetically regulated through gene methylation and hyperacetylation [108,109,110,111]; (2) *ALDH2* expression is regulated by several transcriptional factors [112,113,114] and can be negatively regulated by cAMP, potentially through phosphorylation of its transcription factor hepatocyte nuclear factor 4 by PKA [115]; (3) *ALDH2* is negatively regulated by small non-coding RNAs, like miR-193 and -27a-3p [116,117]; (4) ALDH2 activity can be inactivated by chemical modifications at several functional groups, such as oxidation at Cys residue(s), phosphorylation at Ser residue(s), nitration at Tyr residue(s), and acetylation at Lys residue(s) [104,118]; and (5) ALDH2 activity is regulated by several signaling molecules, including c-Jun N-terminal kinase, AMP-activated protein kinase, ε protein kinase C, and sirtuin 3, through phosphorylating ALDH2 protein or decreasing its acetylation level [119]. 

These mechanisms will have biological effects on ALDH2 function, ultimately contributing to carcinogenesis. Indeed, ALDH2 dysfunction has been widely reported to correlate with tumor initiation and progression in various cancers [120]. Specifically, the ALDH2*2 allele with a slower AcAH-metabolizing capacity represents an increased risk of alcohol-related cancers such as esophageal and head and neck cancers [106,121]. When this slow ALDH2*2 allele is combined with the rapid ADH1B*2/*2 allele (rs1229984), a clear positive dose-response relationship can be seen between alcohol consumption and worse survival in patients with bladder and head and neck cancers [122,123], which may be related to the accumulation of large amounts of AcAH. However, *ADH1B* polymorphisms do no appear to result in significant differences in circulating AcAH levels between *ALDH2* wild-type and heterozygous genotypes [124]. Moreover, it is worth mentioning that the effects of the ALDH2*2 allele on alcohol-related cancers may be complex due to its direct carcinogenesis and indirect protective effect of alcohol avoidance [125].

In addition to the enzymes mentioned above, CYP2E1 also has the ability to oxidize AcAH (Figure 2a) and plays an alternative role in clearing AcAH from the liver after alcohol consumption [126]. The joint effects of ALDH2*2 and *CYP2E1* (e.g., rs2031920) polymorphisms on tumor susceptibility in alcohol drinkers deserve to be evaluated [127].

## 4. Ethanol Metabolism and Its Contribution to Melanoma

As most research on ethanol or AcAH concentrates on the liver and GI tract with little focus on the skin [128,129], we previously summarized their potential roles in skin biology and melanoma [9]. Similarly, despite the well-known roles of ethanol and AcAH metabolizing enzymes, there is a lack of direct evidence for their effects on melanoma. This chapter will examine the expression of these enzymes in melanoma and consider the potential association with melanoma biology.

### 4.1. Potential Roles of Dysfunctional Ethanol and AcAH Metabolism in Melanoma

Ethanol exposure may disrupt a range of melanocyte development processes leading to defective skin pigmentation [130], in which the role of ethanol metabolism is unknown. On the other hand, as previously mentioned in Section 3.1, ethanol increases cAMP/PKA pathway activity, which has been implicated in melanocyte pigmentation, melanomagenesis [131,132], and therapy resistance [133]. Therefore, ethanol may directly affect melanoma initiation and progression. 

The enzymes metabolizing ethanol and AcAH have various polymorphic forms, each with distinct catalytic kinetics, leading to various AcAH production. ALDH2 dysfunction is a significant cause of toxic AcAH accumulation [124]. Additionally, AcAH is produced by normal human microbiota, including those on the skin, and damages DNA, impairs DNA repair, and increases ROS production. High ethanol intake and redox imbalance in ALDH2-related mitochondrial respiration can elevate CYP2E1 levels, causing further increases in ROS levels [73,134]. Excessive ROS production has widespread impacts on cell biology, including inducing inflammation and altering signaling pathways that control cancer stem cell renewal, cell proliferation, differentiation, and angiogenesis, creating a favorable microenvironment for cancer initiation [9]. Evidence suggests that a high pro-oxidant state with impaired antioxidant defenses is related to melanocyte malignant transformation and melanoma progression [135,136]. 

Ethanol consumption directly affects the skin, disrupting skin physiology and homeostasis by influencing cutaneous metabolism, immune response, vasculature, and antioxidant system [137]. This skin damage from ethanol is exacerbated by UV exposure, and ethanol and UV radiation can act synergistically to induce mutations and weaken protection mechanisms, including reducing melanin production and glutathione levels [138]. Ethanol and UV radiation also activate cAMP/PKA, mitogen-activated protein kinase, phosphatidylinositol 3-kinase/protein kinase B, and Wnt/β-catenin signaling pathways, leading to altered expression of the melanocyte transcription factor microphthalmia-associated transcriptional factor (MITF) [9]. MITF regulates melanocyte biology, and its dysregulation is directly linked to melanoma development [139]. 

Animal studies have also shown that ethanol and AcAH promote melanoma progression and metastasis [140]. The exact mechanism is unclear, but ethanol and AcAH may remodel the tumor microenvironment to facilitate tumor migration and invasion. For example, ethanol intake reduces circulating CD8+ T cells and NK cells [141,142], activates the inflammasome [143], induces hypoxia-inducible factor 1 expression [144,145], and enhances extracellular matrix degradation by matrix metalloproteinases [146], all of which contribute to the progression of melanoma.

Overall, dysfunctional ethanol and AcAH metabolism would amplify the impact of ethanol and AcAH in tumor formation. For more information on how ethanol and AcAH contribute to melanoma initiation and progression, please refer to reference [9]. 

### 4.2. Ethanol Metabolizing Enzymes in Human Melanoma

Several recent studies have suggested the potential roles of ethanol metabolizing enzymes in human melanoma by identifying differentially expressed genes using publicly available microarray data. Liu et al. analyzed three microarray datasets from the Gene Expression Omnibus (GEO) database, including GSE15605, GSE46517, and GSE114445, and found that *ADH1B* was significantly downregulated in primary melanoma samples vs. normal skin samples [147]. Their Cox regression analysis revealed that *ADH1B* is an independent prognostic factor in human melanoma [147]. Another study confirmed the downregulation (~9-fold) of *ADH1B* in human primary melanoma samples compared to normal skin tissues by analyzing two of the three datasets (GSE15605 and GSE46517) and another dataset GDS1375 [148]. 

**Figure 3:**
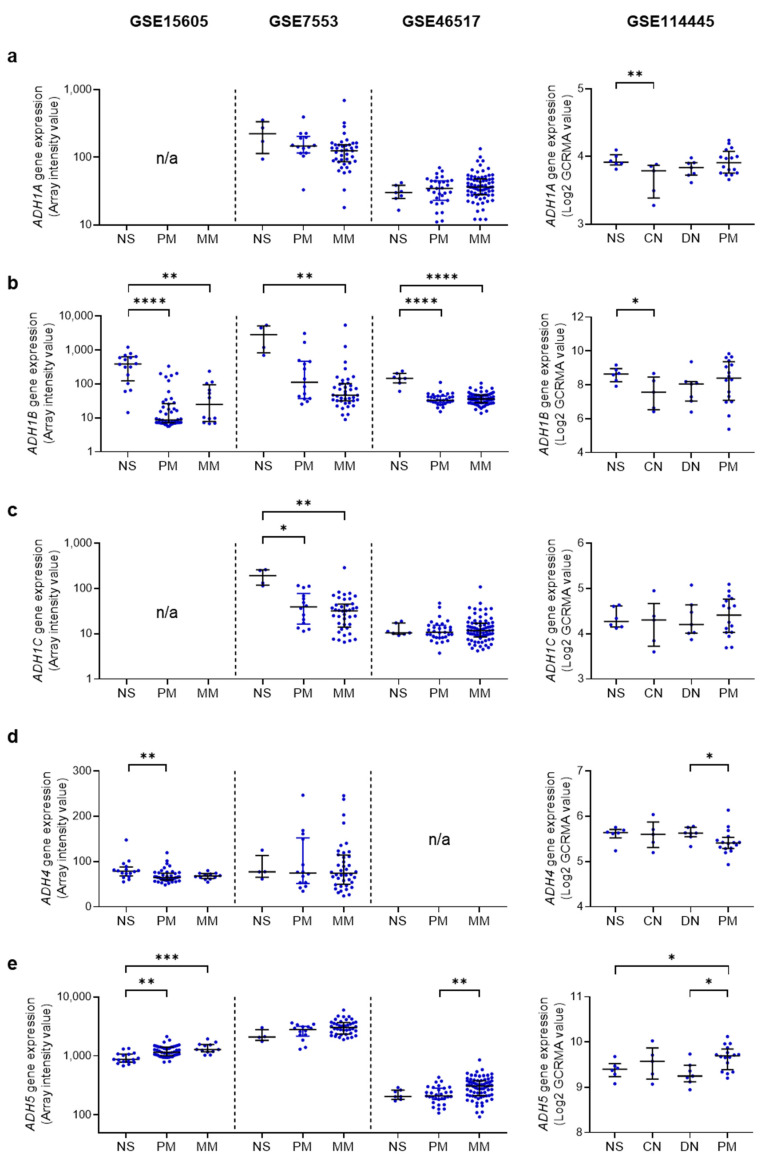

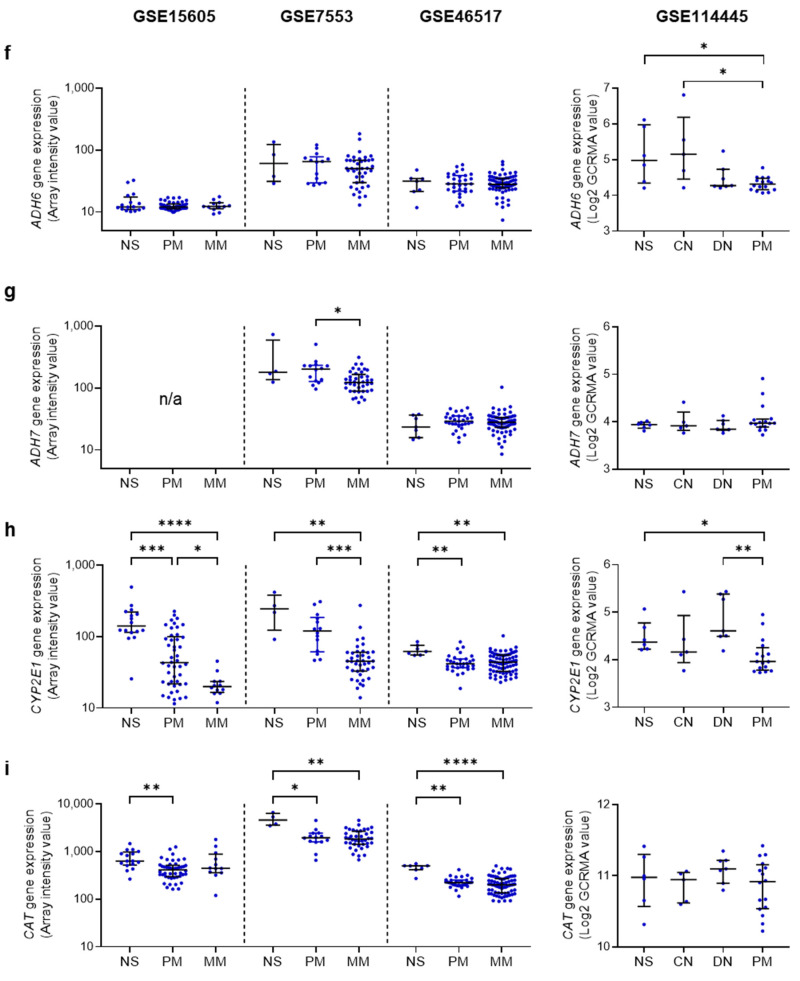
Gene expression of ethanol metabolizing enzymes in normal human skin, melanocytic nevi, and melanoma. Ethanol metabolizing enzyme genes include *ADH1A* (**a**), *ADH1B* (**b**), *ADH1C* (**c**), *ADH4* (**d**), *ADH5* (**e**), *ADH6* (**f**), *ADH7* (**g**), *CYP2E1* (**h**), and *CAT* (**i**). Data from four independent gene profiling studies were analyzed: GSE15605 (16 normal skin (NS) samples, 46 primary melanoma (PM) samples, and 12 metastatic melanoma (MM) samples [149]), GSE7553 (4 NS, 14 PM, and 40 MM [150]), GSE46517 (7 NS, 31 PM, and 73 MM [151]), and GSE114445 (6 NS, 5 common nevus (CN) samples, 7 dysplastic nevus (DN) samples, and 16 PM [152]). In these datasets, if two or more probes were used for a certain gene, the gene expression values of these probes per patient were averaged since they used the same controls. Data are shown as scatter dot plot with median and interquartile range (IQR). * *p* < 0.05, ** *p* < 0.01, *** *p* < 0.001, and **** *p* < 0.0001 (Kruskal–Wallis’s non-parametric test (GSE15605, GSE7553, and GSE46517) and nonparametric estimation of Spearman’s rank correlation (GSE114445)). n/a, not available.

Since the above studies did not include metastatic melanoma samples, we further analyzed the gene expressions of ethanol metabolizing enzymes in the datasets GSE15605, GSE7553, and GSE46517 [149,150,151] (Figure 3a–i, left). Among the ethanol metabolizing enzymes (*ADH1-7*, *CYP2E1*, and *CAT*), *ADH1B*, *ADH5*, and *CAT* were relatively highly expressed in all three datasets. When compared to normal skin tissues, primary melanoma tissues showed reduced *ADH1B*, *CYP2E1*, and *CAT* gene expression in at least two of three datasets (Figure 3b,h,i, left), consistent with previous observations for *ADH1B* [147,148]. Moreover, *CYP2E1* was shown to be further downregulated in metastatic melanoma compared to primary melanoma in two of three datasets (Figure 3h, left). Unexpectedly, *ADH5* was significantly upregulated in primary melanoma samples of one dataset and showed an increasing trend in melanoma in the other two datasets (Figure 3e, left). 

Since melanocytes, the skin pigment-producing cells that give rise to melanoma, make up only a small portion of normal skin tissue [153,154], we further compared the gene expression levels of ethanol metabolizing enzymes in normal human skin, nevus, and melanoma tissues (Figure 3a–i, right). We analyzed another dataset GSE114445, which contained normal skin, common nevi, dysplastic nevi, and primary melanoma [152]. Results showed no differences in the gene expression levels of ethanol metabolizing enzymes between normal skin and melanocytic nevi except for downregulated *ADH1A* and *ADH1B* in common nevi (Figure 3a,b, right). Although we did not find decreased *ADH1B* and *CAT* in primary melanoma compared to normal skin, a decrease in *CYP2E1* and an increase in *ADH5* were observed when compared to normal skin and dysplastic nevi (Figure 3b,e,h,i, right). 

These data suggest a potential role for altered gene expression levels of ethanol metabolizing enzymes in human melanoma. The mechanism behind these data is unknown, but their expression levels may ultimately relate to the production and metabolism of AcAH. 

### 4.3. AcAH Metabolizing Enzymes in Human Melanoma 

Among four AcAH metabolizing enzymes, upregulation of *ALDH1A1* and *ALDH1B1* have been reported in various cancers. ALDH1A1 is involved in cancer stem cell maintenance, metabolism, and drug resistance in multiple cancer types, including melanoma [155]. *ALDH1B1* is upregulated in several cancers, such as colorectal and pancreatic cancers, and acts as an oncogene [156,157]. The role of ALDH1B1 in melanoma remains unclear. On the other hand, *ALDH2* expression has been shown to be downregulated in breast, lung, esophageal, and head and neck cancers, and this reduction in *ALDH2* expression has been linked to a poor prognosis of cancer patients [158,159]. 

**Figure 4:**
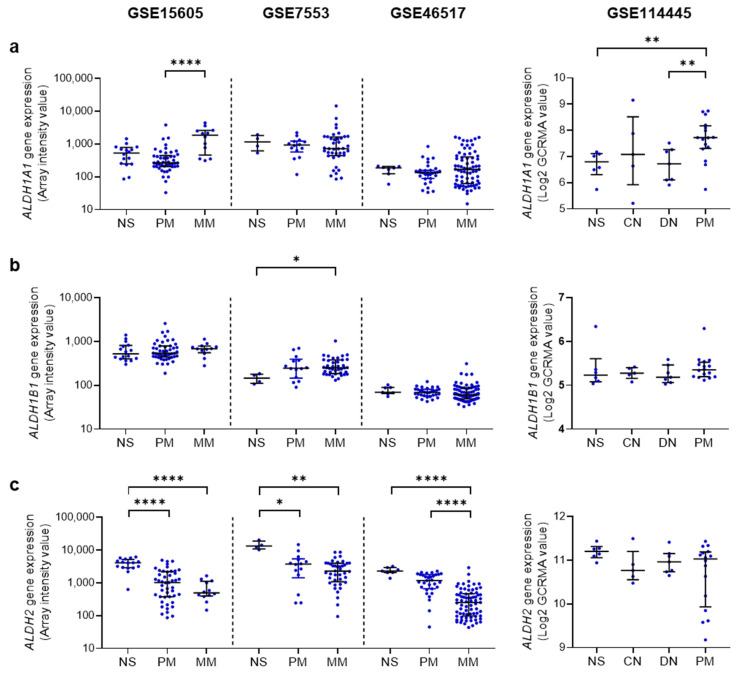
Gene expression of acetaldehyde (AcAH) metabolizing enzymes in normal human skin, melanocytic nevi, and melanoma. AcAH metabolizing enzyme genes include *ALDH1A1* (**a**), *ALDH1B1* (**b**), and *ALDH2* (**c**). See Figure 3 for *CYP2E1*. Data from four independent gene profiling studies were analyzed as described in Figure 3: GSE15605 (16 normal skin (NS) samples, 46 primary melanoma (PM) samples, and 12 metastatic melanoma (MM) samples [149]), GSE7553 (4 NS, 14 PM, and 40 MM [150]), GSE46517 (7 NS, 31 PM, and 73 MM [151]), and GSE114445 (6 NS, 5 common nevus (CN) samples, 7 dysplastic nevus (DN) samples, and 16 PM [152]). Data are shown as scatter dot plot with median and interquartile range (IQR). * *p* < 0.05, ** *p* < 0.01, and **** *p* < 0.0001 (Kruskal–Wallis’s non-parametric test (GSE15605, GSE7553, and GSE46517) and nonparametric estimation of Spearman’s rank correlation (GSE114445)).

We compared the gene expression levels of *ALDH1A1*, *ALDH1B1*, and *ALDH2* in normal human skin vs. primary and metastatic melanoma by analyzing the gene profiling datasets GSE15605, GSE7553, and GSE46517 as explained in Section 4.2 [149,150,151]. We found that *ALDH2* expression levels were significantly downregulated in three GEO melanoma datasets (Figure 4a–c, left). Wu et al. also observed downregulated *ALDH2* gene expression in primary human melanoma [160]. Moreover, analysis of the GSE11445 dataset [152] revealed that some patients with primary melanoma exhibited decreased expression of the *ALDH2* gene compared to normal skin and melanocytic nevi (Figure 4c, right). These findings suggest that the downregulation of *ALDH2* expression could impact clinical outcomes in melanoma patients. 

Considering the consequences of low *ALDH2* expression, leading to AcAH accumulation and compensation by other enzymes, it is plausible to explain how *ALDH2* downregulation in tumor tissues contributes to the poor prognosis of cancer patients. ALDH2 dysfunction has been widely linked to an increased risk of alcohol-related cancers [120], and individuals carrying the ALDH2*2 allele may face a 10-fold increased risk of developing upper esophageal and pharyngeal cancers with chronic alcohol intake [97]. 

However, the clinical significance of *ALDH2* polymorphism in melanoma remains unclear. The ALDH2*2 allele is prevalent in populations with low melanoma incidence, such as Mongolians [161], but rare in high-risk populations like Caucasians with high alcohol consumption [6,161]. Therefore, we analyzed global melanoma data, alcohol consumption, and published *ALDH2* information and found that the wild-type *ALDH2* allele was strongly positively correlated with melanoma incidence (*R* = 0.70; *p* < 0.001), while the allelic variants had a modest to strong negative correlation (*R* = −0.70; *p* < 0.001 and *R* = −0.51; *p* = 0.01 for ALDH2*1/*2 and ALDH2*2/*2, respectively) [6]. Interestingly, alcohol consumption showed similar trends: people with the wild-type *ALDH2* allele tended to drink more alcohol (*R* = 0.39; *p* = 0.07), while the allelic variants consumed less alcohol (*R* = −0.38; *p* = 0.07 and *R* = −0.25; *p* = 0.26 for ALDH2*1/*2 and ALDH2*2/*2, respectively) [6]. These data suggest that carriers of the *ALDH2* mutation may develop less melanoma because they drink less alcohol [105]. This notion is supported by observations by Koyanagi et al. on GI tract cancers [125], who split the *ALDH2* allele effects into the carcinogenic effect (direct effect from increased AcAH exposure) and the protective effect (indirect effect from decreased alcohol intake). They found that while the *ALDH2* allele increased the risk of GI tract cancers, especially those of the upper GI tract, the risk protection was also prominent for all GI tract cancers observed except small intestine cancer [125]. These demonstrate the complexity of the relationship between ALDH2 expression, *ALDH2* polymorphism, and melanoma outcomes. Understanding the mechanisms underlying ALDH2 downregulation or inactivation in cancers not involving *ALDH2* polymorphisms is crucial.

## 5. Conclusions and Future Directions 

Alcohol consumption has long be recognized as a risk factor for many GI tract cancers due to their exposure to mutagenic AcAH. However, growing evidence suggests that alcohol consumption is a potential culprit in cutaneous melanoma. The underlying mechanisms by which ethanol and AcAH induce carcinogenesis in melanoma remain unclear. It is important to investigate whether there are shared mechanisms between melanoma and other cancers. On the other hand, the skin is exposed to toxic environmental metabolites in addition to the ethanol and AcAH that circulate from the digestive system following alcohol drinking and food intake. All these different sources of ethanol and AcAH, combined with other risk factors such as UV radiation and smoking, make the pathogenesis of melanoma complex. Understanding the contributions of each source of ethanol and AcAH in melanoma development is essential. 

A simple but important question is how ethanol and AcAH contribute to melanomagenesis beyond the formation of carcinogenic macromolecular adducts. One possible explanation is that the levels of ethanol and AcAH in the skin exceed the detoxification capacity of metabolic enzymes, thereby activating the cAMP/PKA signaling and promoting the formation of macromolecular adducts. Like the liver, the skin harbors a set of ethanol and AcAH metabolizing enzymes that play a crucial role in preventing the excessive accumulation of ethanol and toxic metabolites. Additionally, the cutaneous antioxidant system can help reduce oxidative stress caused by ethanol metabolism. However, it is still unclear whether there are differences in the biological properties of ethanol and AcAH detoxification and ROS scavenging between skin tissue and the digestive system. 

Nevertheless, evidence supports that downregulation and inactivation of ethanol or AcAH metabolizing enzymes due to genetic polymorphisms and somatic mutations are associated with various human diseases. Therefore, restoring the activity of these metabolic enzymes, particularly the ALDH2 enzyme, may be important to prevent melanoma initiation and progression.
